# Supplementary material for: Enhancement of Mechanical Properties and Bonding Properties of Flake-Zinc-Powder-Modified Epoxy Resin Composites
Source: Polymers (Basel). 2022 Dec 5;14(23):5323. doi: 10.3390/polym14235323 (PMC9740281; doi:10.3390/polym14235323)
Supplement: Supplementary file 1 [file polymers-14-05323-s001.zip › polymers-2053543-supplementary.pdf]

Supplementary Data

Enhancement of mechanical properties and bonding properties of flake zinc powder modified epoxy resin composites

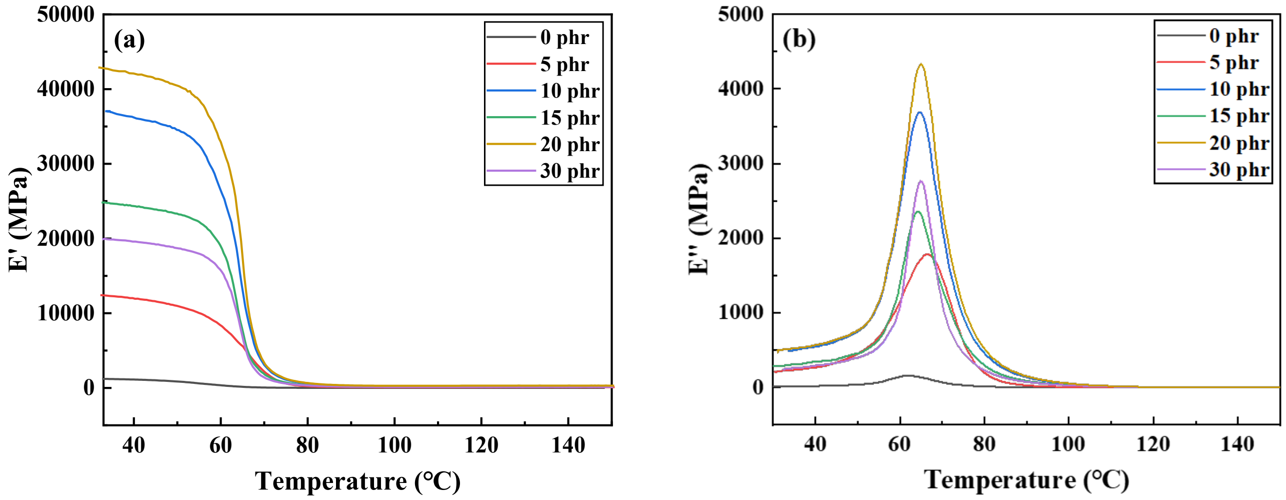

Figure S1. (a) E'; (b) E'' curves of CTPBA/EP epoxy resin modified with different Mf-Zn content
